# Tai Chi for improving motor symptoms in Parkinson’s disease patients: a systematic review of mechanisms

**DOI:** 10.3389/fnagi.2026.1883064

**Published:** 2026-08-19

**Authors:** Zhi Li, Xiupan Wei, Pengyi Gao, Jiayi Zhang, Qian Wang, Pei Wang, Ting Zhou, Hongxing Wang

**Affiliations:** 1 Zhongda Hospital Southeast University, Department of Rehabilitation Medicine, Nanjing, Jiangsu, China; 2 Department of Rehabilitation Medicine, Sichuan Provincial People’s Hospital, School of Medicine, University of Electronic Science and Technology of China, Chengdu, Sichuan, China; 3 Department of Rehabilitation Medicine, West China Hospital, Sichuan University, Chengdu, Sichuan, China

**Keywords:** biomechanics, neural network, neuroinflammation, Parkinson’s disease, Tai Chi

## Abstract

**Background:**

In recent years, Tai Chi (TC) training has played an increasingly important role in the management of motor disorders in Parkinson’s disease (PD) patients. A thorough understanding of the therapeutic mechanisms of TC is crucial for optimizing its clinical efficacy. The long-term practice of TC has been shown to enhance patients’ balance and postural stability, thereby reducing the risk of falls and improving their walking ability.

**Objectives:**

This systematic review seeks to elucidate the mechanisms through which TC enhances motor symptoms in individuals diagnosed with PD. It also endeavors to conduct a comprehensive review of existing evidence pertaining to this phenomenon.

**Methods:**

This systematic review was conducted following PRISMA 2020 guidelines, structured per PICOS (Population: adults with Parkinson’s disease; Intervention: Tai Chi, any style/dosage; Comparator: no intervention, usual care, alternative exercise, or pre-post comparison; Outcomes: biomechanical, molecular/metabolic, or neural-network mechanisms linked to motor symptoms; Design: clinical trials reporting ≥ 1 mechanistic outcome). PubMed, Embase, Cochrane Library, and Web of Science were searched in June 2026, yielding 10 eligible studies (6 RCTs, 4 non-randomized) on TC’s effects in Parkinson’s disease.

**Results and conclusion:**

TC was associated with improvements in postural control, balance, muscle strength, and motor symptoms, alongside reductions in muscle tone and enhancements in quality of life in Parkinson’s patients. Most proposed molecular, metabolic, and neural-network mechanisms rely on indirect (peripheral biomarker) or single-study evidence rather than direct central measurements, and remain hypothesis-generating. Candidate mechanisms include increased dopamine-pathway markers, regulated inflammatory responses, and altered neurotransmitter-related metabolism. TC may also affect amino-acid, energy, and neurotransmitter metabolism – including enhanced arginine biosynthesis, tricarboxylic acid cycle activity, and adenosine levels – and upregulate peripheral blood glutathione, an antioxidant marker that may offset oxidative stress relevant to dopaminergic neuron survival, contributing to its therapeutic effects. Molecularly, TC was linked to enhanced ubiquitin-proteasome system activity in a single study, but this finding is derived from peripheral blood and requires confirmation. Biomechanically, it improves center-of-pressure displacement and muscle activity; at the neural-network level, it is linked to improved coordination and activation in motor-related brain areas. However, these mechanistic conclusions are preliminary. Most molecular and metabolic evidence is indirect (peripheral biomarkers) and derived from single studies with small sample sizes, requiring confirmation in larger, centrally-measured trials.

**Systematic review registration:**

https://www.crd.york.ac.uk/prospero/, identifier CRD42024486254.

## 1 Introduction

Parkinson’s disease (PD) is a neurodegenerative disorder characterized by a prolonged course and gradual progression, leading to worsening functional impairment in patients (Bloem et al., 2021). It is typically associated with dopamine deficiency, along with both motor and non-motor symptoms (Simon et al., 2020). Epidemiological studies indicate that neurological diseases have become a leading cause of disability worldwide, with PD being the fastest-growing neurological disorder (Dorsey et al., 2018). The global prevalence of PD has accelerated due to aging populations (Dorsey et al., 2018), and it has been reported that the global pooled prevalence of Parkinson’s disease is 1.51 cases per 1000 people (Zhu et al., 2024). In addition to its detrimental impact on patients’ quality of life, PD imposes significant healthcare costs (Leite Silva et al., 2023). Currently, the most widely used treatment is levodopa, though symptomatic drug therapy offers limited benefits in certain aspects, and long-term use can lead to complications such as movement disorders (Langeskov-Christensen et al., 2024; Toh, 2013). Research has shown that physical exercise can effectively manage PD and offers potential benefits that may influence clinical practice (Ernst et al., 2023; Langeskov-Christensen et al., 2024). Therefore, the Movement Disorder Society Evidence-Based Medicine Panel recommends exercise as an effective adjunct therapy to levodopa (Mak et al., 2017).

Tai Chi (TC) is a traditional Chinese practice that integrates posture, breathing techniques, and meditation to improve sub-health conditions and alleviate disease symptoms (Song et al., 2020). Unlike high-impact aerobic exercise, Tai Chi emphasises rhythmic weight shifting between the lower limbs, rotational trunk movements, symmetrical foot stepping, and the maintenance of an upright posture throughout, making it inherently low-impact and accessible to older adults and clinical populations (Pan et al., 2018; Wang C. et al., 2022). In the context of PD treatment, long-term and regular TC practice has been shown to effectively enhance motor symptoms (Li G. et al., 2022), improve balance, prevent falls (Li et al., 2012), and boost overall cognitive function (Li et al., 2024; Song et al., 2017). Recently, numerous systematic reviews and meta-analyses have emerged, examining the effects of TC on Parkinson’s patients, particularly its impact on improving quality of life (Navas-Otero et al., 2024), enhancing motor symptoms (Xu et al., 2023; Zhu et al., 2023), and promoting cognitive function (Wang L. et al., 2022; Wang Y. et al., 2022). However, these studies primarily focus on certain aspects of TC’s effectiveness for PD, without fully addressing its potential mechanisms. Some studies have begun to explore the possible mechanisms of TC in other populations, but its specific action in PD remains underexplored. For example, 136 differentially expressed genes were identified in PD patients following TC practice, suggesting that TC influences peripheral immune function in these patients, shedding light on its molecular mechanisms (Yang et al., 2022). Additionally, Li et al. discovered that the improvement in motor symptoms observed in PD patients practicing TC may be attributed to its regulatory effects on metabolism (Li G. et al., 2022).

A clear and comprehensive understanding of the benefits and potential mechanisms of TC in PD is crucial for assessing its value as a complementary and adjunctive treatment. Currently, the evidence for specific mechanisms, particularly central dopaminergic restoration, remains indirect and preliminary. Therefore, we conducted a thorough review to summarize mechanistic studies on the efficacy of TC as a supportive therapy for Parkinson’s patients. To ensure methodological rigor and reproducibility, this review was conducted and is reported in accordance with the PRISMA 2020 statement (Page et al., 2021), and the review question was structured *a priori* using the PICOS framework. Our aim is to clarify the relationship between specific interventions and various outcomes, provide insights for adjusting and improving treatment protocols, optimize therapeutic effects, and facilitate the translation of research findings into clinical practice.

## 2 Methods

### 2.1 Protocol and registration

This systematic review was conducted and is reported in accordance with the Preferred Reporting Items for Systematic Reviews and Meta-Analyses (PRISMA 2020) statement (Page et al., 2021). The review protocol was prospectively registered on PROSPERO (registration number CRD42024486254) prior to full-text screening and data extraction.

### 2.2 Research question (PICOS)

The review question was defined *a priori* using the PICOS framework, as follows:

Population (P): adults with a clinical diagnosis of idiopathic Parkinson’s disease, of any age, sex, or Hoehn and Yahr disease stage. Intervention (I): Tai Chi practiced in any style, dosage, or duration, whether alone or combined with Qigong-specific breathing components. Comparator (C): no intervention, usual/standard care, an alternative exercise modality, a waitlist control, or a within-subject pre-post (single-group) comparison; a comparator was not required for inclusion given the mechanistic focus of this review. Outcomes (O): any biomechanical, molecular/metabolic, or neural-network measure proposed as a mechanism underlying TC’s effect on PD motor symptoms, together with any co-reported clinical motor outcome (e.g., UPDRS-III, gait, balance). Study design (S): clinical trials (randomized or non-randomized, including single-arm before-after designs) reporting at least one mechanistic outcome; reviews, protocols, case reports, letters, comments, clinical practice guidelines, and non-peer-reviewed conference material were excluded.

Accordingly, the guiding review question was: ‘Through what biomechanical, molecular/metabolic, and neural-network mechanisms does Tai Chi improve motor symptoms in adults with Parkinson’s disease, as evidenced by clinical trials?’

### 2.3 Search strategy

We conducted a systematic literature search of articles published between 2005 and 2026. The search was performed in English. A comprehensive search strategy was executed in the PubMed, Embase, Cochrane Library, and Web of Science Core Collection databases. Keywords included “Parkinson Disease,” “Idiopathic Parkinson’s Disease,” “Lewy Body Parkinson’s Disease,” and “Tai Chi” or “Tai-ji.” The complete, reproducible search strategy for all four databases is presented in Table 1 below. Additionally, other methods such as reviewing the reference lists of relevant identified studies were employed. All searches were re-run on 12 June 2026 to confirm the final search date, and de-duplication was performed, followed by manual verification.

**TABLE 1a:** Complete search strategy – Web of Science.

Item	Search terms
#1	TS = (tai ji)
#2	((((((ALL = (Tai-ji)) OR ALL = (Tai Chi)) OR ALL = (Chi, Tai)) OR ALL = (Tai Ji Quan)) OR ALL = (Quan, Tai Ji))) OR ALL = (taiji)) OR ALL = (T’ai Chi)) OR ALL = (Tai Chi Chuan)
#3	(#1) OR #2
#4	TS = (Parkinson Disease)
#5	(((((((((((ALL = (Idiopathic Parkinson’s Disease)) OR ALL = (Lewy Body Parkinson’s Disease)) OR ALL = (Parkinson’s Disease, Idiopathic)) OR ALL = (Parkinson Disease)) OR ALL = (Parkinson’s Disease, Lewy Body)) OR ALL = (Parkinson Disease, Idiopathic)) OR ALL = (Parkinson’s Disease)) OR ALL = (Idiopathic Parkinson Disease)) OR ALL = (Lewy Body Parkinson Disease)) OR ALL = (Primary Parkinsonism)) OR ALL = (Parkinsonism, Primary)) OR ALL = (Paralysis Agitans)
#6	(#5) OR #4
#7	(#6) AND #3

**TABLE 1b:** Complete search strategy – PubMed.

	Search terms
#1	(((((((((((((Parkinson Disease[Title/Abstract]) OR (Idiopathic Parkinson’s Disease[Title/Abstract])) OR (Lewy Body Parkinson’s Disease[Title/Abstract])) OR (Parkinson’s Disease, Idiopathic[Title/Abstract])) OR (Parkinson’s Disease, Lewy Body[Title/Abstract])) OR (Parkinson Disease, Idiopathic[Title/Abstract])) OR (Parkinson’s Disease[Title/Abstract])) OR (Idiopathic Parkinson Disease[Title/Abstract])) OR (Lewy Body Parkinson Disease[Title/Abstract])) OR (Primary Parkinsonism[Title/Abstract])) OR (Parkinsonism, Primary[Title/Abstract])) OR (Paralysis Agitans[Title/Abstract])) OR (Parkinson Disease[MeSH Terms])) AND (((((((((((Tai-ji[Title/Abstract]) OR (Tai Chi[Title/Abstract])) OR (Chi, Tai[Title/Abstract])) OR (Tai Ji Quan[Title/Abstract])) OR (Ji Quan, Tai[Title/Abstract])) OR (Quan, Tai Ji[Title/Abstract])) OR (Taiji[Title/Abstract])) OR (Taijiquan[Title/Abstract])) OR (T’ai Chi[Title/Abstract])) OR (Tai Chi Chuan[Title/Abstract])) OR (“Tai Ji”[Mesh]))

**TABLE 1c:** Complete search strategy – Embase.

Item	Search terms
#1	(((((((((((((‘parkinson disease’:ti,ab,kw) OR (‘idiopathic parkinson‘s disease’:ti,ab,kw)) OR (‘lewy body parkinson’s disease’:ti,ab,kw)) OR (‘parkinson‘s disease, idiopathic’:ti,ab,kw)) OR (‘parkinson’s disease, lewy body’:ti,ab,kw)) OR (‘parkinson disease, idiopathic’:ti,ab,kw)) OR (‘parkinson’s disease’:ti,ab,kw)) OR (‘idiopathic parkinson disease’:ti,ab,kw)) OR (‘lewy body parkinson disease’:ti,ab,kw)) OR (‘primary parkinsonism’:ti,ab,kw)) OR (‘parkinsonism, primary’:ti,ab,kw)) OR (‘paralysis agitans’:ti,ab,kw)) OR (‘Parkinson disease’/exp)) AND (((((((((((‘tai-ji’:ti,ab,kw) OR (‘tai chi’:ti,ab,kw)) OR (‘chi, tai’:ti,ab,kw)) OR (‘tai ji quan’:ti,ab,kw)) OR (‘ji quan, tai’:ti,ab,kw)) OR (‘quan, tai ji’:ti,ab,kw)) OR (‘taiji’:ti,ab,kw)) OR (‘taijiquan’:ti,ab,kw)) OR (‘t’ai chi’:ti,ab,kw)) OR (‘tai chi chuan’:ti,ab,kw)) OR (‘Tai Chi’/exp))

**TABLE 1d:** Complete search strategy – EBM Reviews – Cochrane Central Register of Controlled Trials, it is a specific database version provided on the Ovid platform, and its core content is the globally recognized, high-quality Cochrane Central Register of Controlled Trials.

Item	Search terms
#1	(Idiopathic Parkinson‘s Disease or Lewy Body Parkinson‘s Disease or Parkinson’s Disease, Idiopathic or Parkinson‘s Disease, Lewy Body or Parkinson Disease, Idiopathic or Parkinson‘s Disease or Idiopathic Parkinson Disease or Lewy Body Parkinson Disease or Primary Parkinsonism or Parkinsonism, Primary or Paralysis Agitans).ab.
#2	(Tai-ji or Tai Chi or Chi, Tai or Tai Ji Quan or Ji Quan, Tai or Quan, Tai Ji or Taiji or Taijiquan or T’ai Chi or Tai Chi Chuan).ab.
#3	1 and 2

### 2.4 Selection criteria

The inclusion criteria were as follows: 1) the study type was limited to clinical trials; 2) all studies must include PD patients, regardless of age or disease stage; 3) the clinical trials had to incorporate mechanistic studies; 4) any form of TC had to be included as an intervention for PD patients; 5) the studies were published in English; and 6) the study was published before June 12, 2026. We excluded reviews, protocols, case reports, letters, comments, and clinical practice guidelines. We also excluded conference abstracts, posters, and other non-peer-reviewed proceedings, as these typically lack sufficient methodological detail for mechanistic interpretation and had not undergone full peer review. Irrelevant data and studies with unavailable full texts were also excluded. Two authors independently completed the screening process, with any disagreements resolved through discussion.

### 2.5 Methodological quality assessment

The methodological quality of included studies was assessed using two validated tools. Randomized controlled trials (*n* = 6) were evaluated using the Cochrane Risk of Bias 2.0 (RoB 2.0) tool across five domains. The quality of the four included non-randomized studies (*n* = 4) was assessed using both the Risk of Bias In Non-randomized Studies of Interventions (ROBINS-I) tool.

For the randomized controlled trials (RCTs), quality was evaluated using the Cochrane Risk of Bias 2.0 (RoB 2.0) tool, which appraises five domains: (1) bias arising from the randomization process; (2) bias due to deviations from the intended interventions; (3) bias due to missing outcome data; (4) bias in the measurement of the outcome; and (5) bias in the selection of the reported result. Each domain was judged as low risk, some concerns, or high risk, and an overall judgment was assigned accordingly (Sterne et al., 2019).

### 2.6 Data extraction

Two reviewers independently extracted efficacy data on TC’s impact on motor symptoms in PD, as well as related mechanistic studies, from the included trials. Any discrepancies in evaluation scores were resolved through discussion. Key aspects of the studies, such as study design, group setting, intervention setting, facilitator/delivery, and intervention description, were summarized in Supplemental material 1, and a consolidated central evidence table (Supplemental material 2) synthesizing sample size, PD stage, TC protocol, comparator, mechanistic outcomes, main findings, and risk-of-bias rating for every included study is presented.

### 2.7 Data synthesis

Since there is no standardized protocol or analysis method for evaluating the mechanisms of TC’s effects on PD, methodologies varied across studies. Due to the heterogeneity among studies, an overall meta-analysis and quantitative synthesis were not feasible. Therefore, results were assessed using simple descriptive statistics and presented in a narrative synthesis. Findings were categorized and described based on the type of treatment mechanism. This review focuses on the effects of TC on motor symptoms in PD.

## 3 Results

### 3.1 Study selection

**FIGURE 1:**
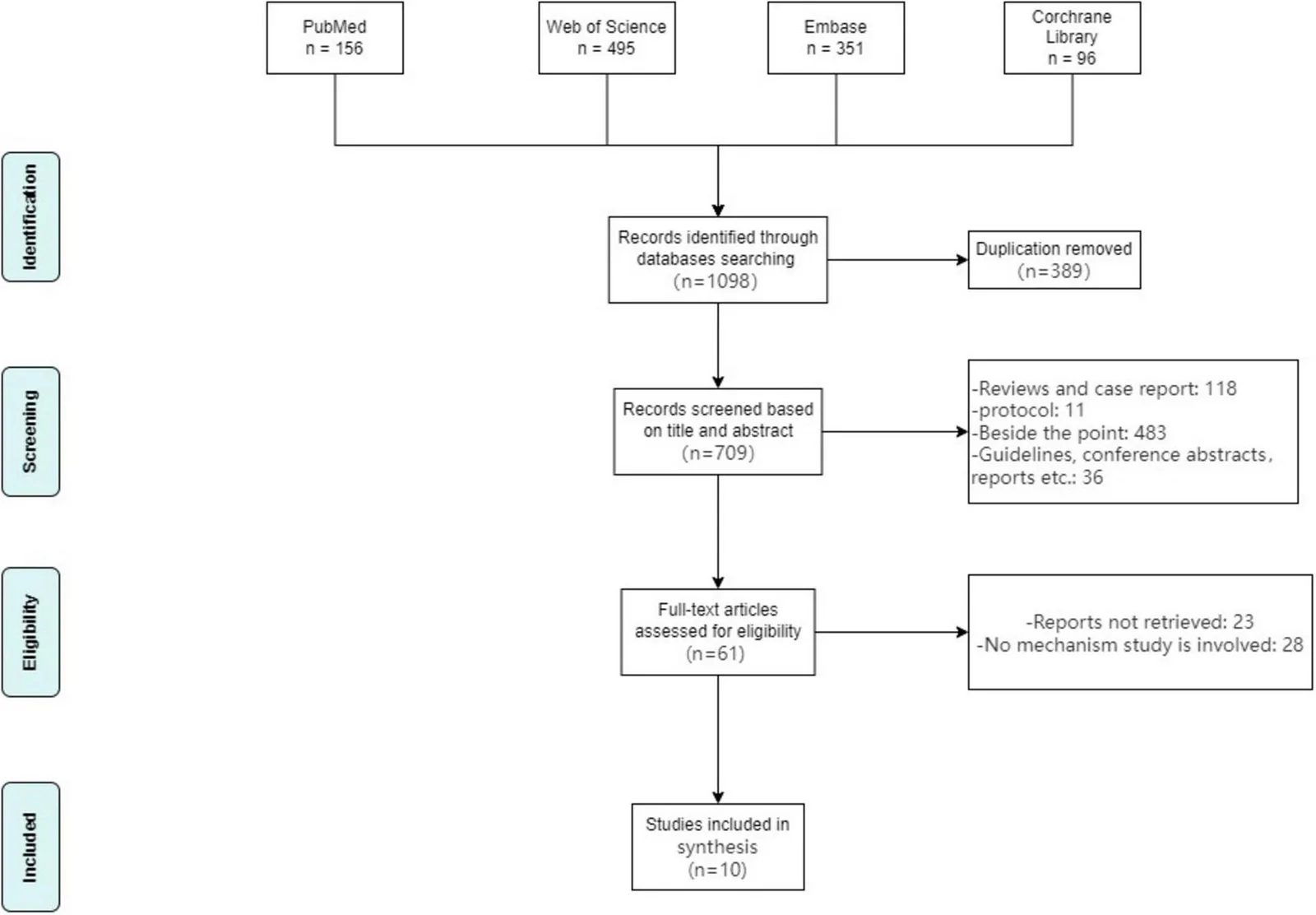
Flow diagram of study selection.

A total of 1098 articles were identified through the literature search from the following databases: PubMed (*N* = 156), Web of Science (*N* = 495), Embase (*N* = 351), Cochrane Library (*N* = 96). After removing 389 duplicate records and screening 709 studies based on titles and abstracts, 10 studies met the inclusion criteria and were included in this review. The full texts of these 10 studies were read (Figure 1).

Other major reasons for excluding studies included: the study’s purpose did not involve TC and its therapeutic mechanisms, the subjects were not Parkinson’s patients, the article format was guidelines, case reports, reviews, posters, or conference reports, or the full text was not available. Ultimately, 10 studies were selected for this review. Supplemental material 1 provides a summary of all the included studies.

### 3.2 Methodological quality of the studies

**FIGURE 2:**
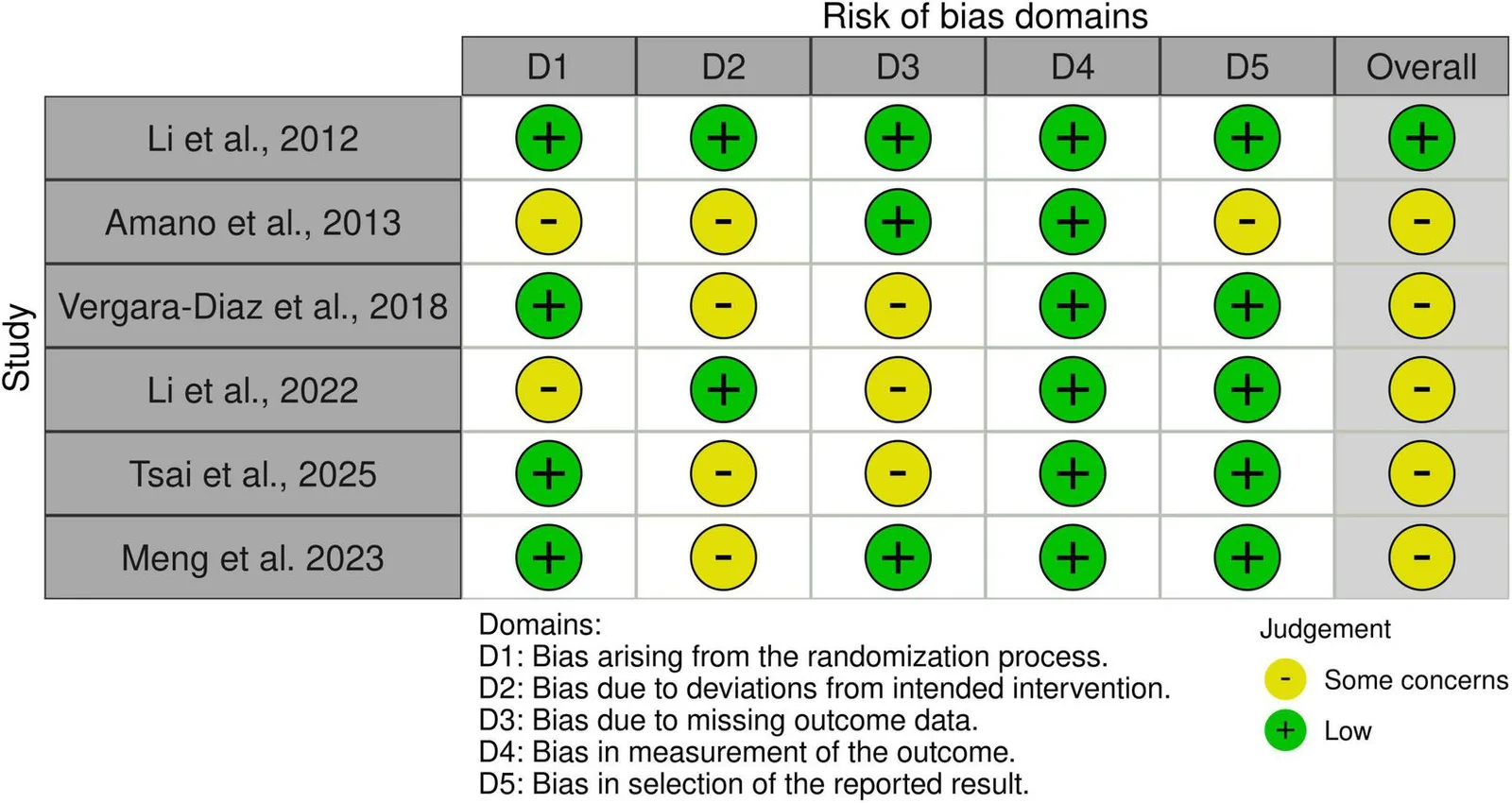
Risk of bias assessment of included randomized controlled trials using the Cochrane Risk of Bias 2.0 (RoB 2.0) tool.

The methodological quality of the six RCTs was assessed using the Cochrane RoB 2.0 tool, and the results are presented in Figure 2. Overall, one study was judged as having a low risk of bias, while the remaining studies were rated as having some concerns. No study was considered to have a high overall risk of bias. Most studies had a low risk of bias in outcome measurement and selection of reported results. Some concerns were mainly related to insufficient reporting of allocation concealment, missing outcome data, and deviations from the intended intervention. Given the behavioral nature of TC interventions, lack of participant blinding was not considered sufficient to indicate bias due to deviations from intended interventions. Sample size was not considered in the RoB 2 judgement.

Regarding individual domains, most studies adequately generated random sequences but lacked detailed concealment descriptions. Blinding of participants and therapists was unfeasible in exercise trials, yet this alone did not determine bias judgments; assessments instead considered protocol adherence and intention-to-treat analyses. Attrition was balanced and low in most studies; higher dropout rates led to some concerns in a few. Outcome measurement was generally low risk due to objective assessments and blinded evaluators, while selective reporting concerns arose mainly from inadequate trial registration details. In conclusion, the included trials showed low-to-moderate risk of bias, with limitations largely reflecting incomplete methodological reporting rather than evidence of systematic bias.

Reassessment of the four non-randomized studies using the ROBINS-I tool resulted in an overall judgment of serious risk of bias for all studies. This discrepancy can be attributed to several factors. First, all four studies employed uncontrolled single-arm before–after designs without concurrent control groups. Although this fundamental design limitation is not adequately reflected, ROBINS-I identifies it as a major source of serious bias due to confounding. Second, (Law and Li, 2023) was judged to have a serious risk of bias due to deviations from intended interventions, as participants engaged in additional physical activities, including boxing, during the intervention period. Third, (Yang et al., 2022) was rated as having a serious risk of bias in the selection of the reported result because its highly exploratory weighted gene co-expression network analysis (WGCNA) and hub-gene identification procedures were conducted without a prespecified or preregistered statistical analysis plan. The results of the quality assessment are presented in Table 2.

**TABLE 2:** Study quality using the ROBINS-I.

Study	1	2	3	4	5	6	7	Total
Kim et al., 2014	Serious	Low	Low	Moderate	Low	Low	Moderate	Serious
Kim et al., 2011	Serious	Low	Low	Moderate	Low	Moderate	Moderate	Serious
Law and Li, 2023	Serious	Low	Low	Serious	Low	Moderate	Moderate	Serious
Yang et al., 2022	Serious	Moderate	Moderate	No information	Moderate	Low	Serious	Serious

1: Confounding; 2: Selection bias; 3: Bias in measurement classification of interventions; 4: Bias due to deviations from intended interventions; 5: Bias due to missing data; 6: Bias in measurement of outcomes; 7: Bias in selection of the reported result.

### 3.3 Characteristics of included studies

Figure 1 illustrates the research flow throughout the review process. A total of 6 studies employed randomized controlled trial designs, while the remaining 4 used other trial designs. The review included 513 participants, with sample sizes ranging from 10 to 195 across the studies.

Among the 10 studies, there were variations in the TC intervention protocols and duration. The intervention periods ranged from 12 to 24 weeks, with each session lasting at least 50 minutes. And six of the included studies adopted Yang-style Tai Chi, the most widely practiced form internationally, which is characterized by slow, flowing movements with an emphasis on weight shifting and postural control. The Tai Chi styles used in the remaining studies were not explicitly stated by the original authors. Regarding Qi Gong, two studies explicitly reported incorporating Qi Gong-specific breathing techniques into their training protocols. The studies evaluated various outcomes, including brain function imaging indicators, blood-related biomarkers, gait, and balance measures. Specific assessment tools included the Single Leg Standing (SLS) test, Timed Up and Go (TUG) test, Unified PD Rating Scale – Motor Section (UPDRS-III), Montreal Cognitive Assessment (MoCA), PD Questionnaire-39 (PDQ-39), Berg Balance Scale (BBS), and other motor performance assessments.

### 3.4 Effects of TC on balance

Multiple studies have demonstrated the beneficial effects of TC on balance in patients with PD. Similarly, TC alone has been shown to enhance balance in Parkinson’s patients (Li et al., 2012; Li G. et al., 2022). Other studies have investigated the effects of combining TC with action observation training (AOT) (Meng et al., 2023) or dual-task TC training (Vergara-Diaz et al., 2018), both of which also resulted in improvements in balance. Among the included studies, only one trial (Li et al., 2012) conducted a 3-month post-intervention follow-up and reported maintenance of balance and gait improvements. All other studies lacked post-intervention follow-up assessments. Therefore, the persistence of TC’s effects after training cessation remains largely unexplored, and the single positive follow-up study requires replication.

### 3.5 Effects of TC on gait

TC exercise has been shown to improve dynamic postural control in patients with mild to moderate PD, thereby ameliorating balance dysfunction, movement disorders, hypokinesia, and tremor or discontinuous movement (Amano et al., 2013). Gait improvements are significant, evidenced by increased rear foot stride length (Law and Li, 2023). One study observed that dual-task TC intervention improved gait stride time variability, highlighting TC’s benefits in optimizing gait performance (Vergara-Diaz et al., 2018). Additionally, evaluations using gait analysis and balance test systems demonstrated that TC training enhances gait by increasing stride length and speed while reducing stride variability. However, some studies have found TC ineffective in improving gait initiation, gait performance, or reducing disability rates in Parkinson’s patients, possibly due to variations in the definitions of relevant research indicators (Amano et al., 2013).

### 3.6 Effects of TC on quality of life and activities of daily living (ADL)

Research indicates that TC practice can significantly enhance the quality of life for PD patients. It effectively improves mood, overall physical and mental functions, and is particularly beneficial in alleviating depression and anxiety. These improvements are attributed to TC’s ability to enhance limb function, balance, and walking ability in PD patients, reduce the severity of motor symptoms, and decrease muscle tension. Supplemental material 2 summarizes – for every included study – the sample size, PD stage, Tai Chi protocol, comparator, mechanistic outcome(s), main findings, and risk-of-bias rating.

## 4 Discussion

Mechanistic findings were classified into three levels of evidence: (1) direct evidence, defined as outcomes measured within the central nervous system or directly along the proposed pathway (e.g., functional magnetic resonance imaging(fMRI) connectivity in patients with Parkinson’s disease); (2) indirect evidence, defined as peripheral or surrogate biomarkers (e.g., circulating cytokines, leukocyte gene expression, or serum metabolites) that are biologically plausible correlates of central processes but were not measured within the central nervous system; and (3) speculative mechanisms, defined as interpretations extrapolated from a single study, animal or non-Parkinson’s disease human models, or theoretical pathways that were not directly evaluated in the included studies. Unless otherwise specified, most molecular and metabolic findings are classified as indirect evidence or speculative mechanisms and should therefore be interpreted as hypothesis-generating rather than as evidence supporting established mechanisms, such as restoration of central dopaminergic function or neural network remodeling.

### 4.1 Biomechanical mechanisms

During daily walking tasks or when crossing obstacles, the human body controls the center of mass (COM) by adjusting the movement of the center of pressure (COP) in the anterior-posterior (A-P) and medial-lateral (M-L) directions to maintain dynamic postural stability (Bax et al., 2020). The swing limb’s hip abductor muscles also contribute to lateral COP movement (Rogers and Pai, 1990). At the onset of walking, the COP must move backward to generate forward momentum and initiate the task (Polcyn et al., 1998). TC training has been shown to increase the amplitude of COP displacement in both the A-P and M-L directions, thereby enhancing the mechanisms for generating momentum and maintaining balance and lateral stability (Kim et al., 2011, 2014). Improved COP activity in the A-P direction is linked to efficient activation of the tibialis anterior muscle (Halliday et al., 1998) and restored anticipatory postural adjustments. Additionally, increased COP displacement in the M-L direction results from enhanced hip muscle strength (Winter et al., 2003), and this may be related to TC training improving the neuromuscular function of the gluteus medius, tensor fasciae latae, and tibialis anterior in Parkinson’s patients. Consistent with these studies, TC exercise enhances dynamic postural stability in Parkinson’s patients by increasing step length of the hind limbs, enhancing ankle abduction of the forelimbs, and improving control of COM and COP movements in the A-P and M-L directions during exercise (Law and Li, 2023).

However, a study (Amano et al., 2013) has indicated that TC training does not increase posterior or lateral displacement of the COP (i.e., in the direction of the swinging limbs), which may be attributed to variations in the definition and measurement of COP displacement across studies. Additionally, there were no significant improvements observed in swing area and Functional Reach Test (FRT), and the enhancements in gait performance and balance ability were not pronounced (Amano et al., 2013). These inconsistencies may be related to unclear measurement methods and definitions of COP displacement.

Generally, there are two primary strategies for recovering balance after a fall: the ankle strategy, which involves rotating the center of mass around the feet, and the hip strategy, which involves rotating body parts in the opposite direction (van Schooten et al., 2024). TC emphasizes shifting the center of gravity and ankle swaying to effectively move the center of gravity to the edge of stability. By improving participants’ ability to use effective swaying strategies (either at the ankle or hip joint), TC enhances their ability to perform controlled movements near the limit of stability, thereby improving balance control and reducing movement disorders and enhancing ADL (Li et al., 2012). Continuous shifting of the center of gravity increases proprioceptive input and core control (Rizzato et al., 2024). Overall, the biomechanical mechanisms identified above are supported chiefly by direct kinematic evidence derived from the included studies themselves. During TC training, the body’s center of gravity moves slowly up, down, left, and right between the feet, while the support surface’s size and position are altered by constantly changing foot placement.

### 4.2 Molecular mechanisms

Huntingtin-interacting protein 2 (HIP2) is an E2 ubiquitin-binding enzyme linked to neurodegenerative diseases. In PD models, reduced HIP2 expression can impair motor symptoms and increase susceptibility to dopaminergic degeneration (Su et al., 2018). Abnormal expression of the E2 ubiquitin-binding enzyme can lead to ubiquitination disorders, disrupting the ubiquitin-proteasome system. This disruption prevents effective degradation of abnormal α-synuclein, leading to its aggregation and increased toxicity, which accelerates neuronal degeneration and death (Opattova et al., 2015). A study (Li G. et al., 2022) has found that TC training can reverse the downregulation of HIP2 mRNA in PD patients, a change associated with improved motor symptoms following TC training. This finding is based on a single study measuring HIP2 mRNA expression in peripheral blood, an indirect surrogate rather than a direct measure of central nervous system processes. It should therefore be interpreted as hypothesis-generating, suggesting that TC may reduce susceptibility to dopaminergic degeneration through modulation of the ubiquitin–proteasome system rather than establishing this mechanism (Li G. et al., 2022).

Inflammation plays a crucial role in the pathogenesis and progression of PD (Jiang et al., 2018). Lipopolysaccharide (LPS) induces Parkinson’s symptoms through IL-1β, suggesting that IL-1β may contribute to the onset or progression of the disease (Saghazadeh et al., 2016). TC training has been shown to reduce IL-1β levels in Parkinson’s patients, thereby improving motor symptoms (Li G. et al., 2022). Meta-analyses indicate that Parkinson’s patients have higher concentrations of peripheral inflammatory cytokines, such as IL-6, TNF, IL-1β, IL-2, IL-10, and C-reactive protein, compared to healthy controls (Qin et al., 2016). Bioinformatics studies suggest that TC affects peripheral immunity in Parkinson’s patients by modulating genes involved in neutrophil activation, T cell activation, NOD-like receptors, and IL-17 signaling pathways (Yang et al., 2022). These cytokine and gene-expression changes are peripheral biomarkers, each reported in a single study; they are biologically plausible correlates of central neuroinflammation but do not themselves demonstrate a central anti-inflammatory effect of TC in PD.

L-arginine is crucial for the synthesis of nitric oxide, which influences oxidative stress and energy metabolism, playing a key role in the pathogenesis of PD (Virarkar et al., 2013). Deficiencies in TCA (tricarboxylic acid) cycle enzymes and mitochondrial dysfunction have also been observed in PD (Bose and Beal, 2016). Studies have shown that TC training affects various metabolic pathways, particularly amino acid metabolism, energy metabolism, and neurotransmitter metabolism. Improvements in amino acid metabolism (such as L-arginine and arginine biosynthesis) and energy metabolism (including the TCA cycle and long-chain fatty acid oxidation) were reported in a single peripheral-metabolomics study and may contribute to neuroprotection and enhancements in motor symptoms, though this remains to be confirmed (Li G. et al., 2022).

The primary motor symptoms of PD include rigidity, bradykinesia, and resting tremor, with rigidity and bradykinesia being associated with dopamine deficiency (Niemi et al., 2024). Additionally, tyrosine hydroxylase, a rate-limiting enzyme in dopamine synthesis, plays a critical role in hydroxylation (Shao et al., 2024). TC increases serum tyrosine hydroxylase (TH) content in PD patients, a peripheral (indirect) marker that may create favorable conditions for DA synthesis, though serum TH does not directly demonstrate central dopaminergic change. Moreover, early changes in DA presynaptic mechanisms are indicated by a gradual increase in cerebrospinal fluid HVA concentration, which may be related to movement disorders in PD (Stefani et al., 2017).

### 4.3 Metabolic mechanisms

Long-term Tai Chi training has been reported to be associated with remodeled amino-acid, energy, and neurotransmitter metabolism in Parkinson’s disease in one study. These changes, including enhanced arginine biosynthesis, TCA cycle activity, and adenosine levels, were correlated with improvements in motor symptoms (UPDRS scores), suggesting, though not establishing, that metabolic modulation contributes to the therapeutic benefit of Tai Chi (Li G. et al., 2022). Replication in independent cohorts is needed before this association can be considered a confirmed mechanism.

Research has indicated that the mechanism by which Tai Chi exercise may improve motor symptoms in patients with PD primarily involves upregulating peripheral blood glutathione (GSH) levels, thereby potentially enhancing the body’s antioxidant defense capacity, which could plausibly alleviate oxidative stress-induced damage to dopaminergic neurons and motor-related muscles, thereby, hypothetically, delaying motor function deterioration while improving balance and strength (Tsai et al., 2025). As GSH was measured only in peripheral blood in a single trial, this pathway remains indirect evidence and awaits confirmation with central or longitudinal biomarkers.

### 4.4 Neural network mechanisms

Research on TC’s effects on the brain network in the elderly has shown initial positive results. Using resting-state fMRI, studies have observed enhanced connectivity within the default mode network (DMN). A similar approach was employed to investigate TC’s impact on the brain network in PD patients. This research found that changes in DMN connectivity correlated with improvements in both the UPDRS total score and UPDRS-III score (Li G. et al., 2022). The connectivity between the precuneus and motor areas in the DMN is thought to be linked to motor mental imagery and planning processes (Thibes et al., 2017). Thus, the improvement in DMN connectivity may plausibly contribute to the observed enhancements in motor symptoms in Parkinson’s patients following TC training, although this remains a correlational, single-study finding rather than established causal evidence.

Additionally, the study noted increased functional connectivity in the visual network (VN) area. Since visual proprioceptive conflict affects gait and balance, visual cues can help reduce vestibular noise and improve balance (Assländer et al., 2015). Parkinson’s patients typically exhibit reduced functional connectivity in the VN area (Ruan et al., 2020), and TC appears to be associated with connectivity in this region in this single study. Other studies have explored TC’s effects on the DMN in Parkinson’s patients, combining TC with action observation training (AOT). This research found increased functional connectivity between the dorsomedial prefrontal cortex (dmPFC) and cerebellum crus 1, right posterior inferior parietal lobule (pIPL), supramarginal cortex, and the temporoparietal junction (TPJ), extending to the occipital and temporal gyrus (Meng et al., 2023). Activation of the TPJ, along with the right pIPL and supramarginal cortex, is associated with movement observation (Aracil-Bolaños et al., 2019). TC, as a physical exercise, can induce changes in frontal cortex plasticity, with enhanced plasticity in this region being significantly associated with improved balance ability (Li J. et al., 2022).

Studies have demonstrated that performing cognitive tasks while walking can interfere with motor control, as these tasks compete for shared neural resources. In PD, the increased motor control costs associated with cognitive tasks may be partly due to reduced cortical resources and errors in resource allocation. TC training, especially when combined with action observation training (AOT), can affect cognitive-motor interactions in PD, with one study reporting better coordination between relevant brain areas (Vergara-Diaz et al., 2018). Additionally, TC exercise was reported, in a single study, to significantly impact regional brain coherence (ReHo) in Parkinson’s patients. Changes in ReHo values in specific brain areas, such as the infraorbital gyrus and lingual gyrus, indicate adjustments in neural activity.

Previous research has indicated that functional connectivity in the brains of PD patients is significantly weakened, particularly in the cortico-basal ganglia loop and the mesolimbic system-basal ganglia loop (Luo et al., 2014). Exercise has been shown to significantly enhance resting-state brain connectivity between the medial prefrontal lobe and the medial temporal lobe in healthy elderly individuals (Li et al., 2014). Building on this, some studies have explored the effects of TC on brain function using fMRI. These studies suggest that TC may help counteract the decline in brain connectivity observed in PD patients. It appears to gradually be associated with strengthened functional connections between several brain regions, including the frontal and temporal lobes, supplementary motor area, basal ganglia, and cerebellum (again, based on a small number of neuroimaging studies). These areas are typically associated with cognitive and motor symptoms.

These mechanisms are supported by direct neuroimaging evidence; however, they were derived from only 2 of the 10 included studies, and the findings should not be generalized to all TC protocols.

## 5 Conclusions

This study suggests that TC may have a positive effect on improving motor symptoms in patients with PD, supported mainly by direct biomechanical and preliminary neuroimaging evidence. It notably enhances balance, gait, and reduces the risk of falls, thereby improving patients’ quality of life and their ability to perform activities of daily living. TC training is associated with therapeutic benefits during the intervention period; however, evidence for lasting benefits after training cessation remains limited, with candidate, largely indirect mechanisms including markers consistent with increased dopamine pathway activity, modulation of inflammatory responses, and changes in neurotransmitter-related metabolism. On a molecular level, TC may be associated with enhanced function of the ubiquitin-proteasome system. Biomechanically, it improves center of pressure (COP) displacement and enhances the activity of related muscle groups (direct evidence). At the neural network level, TC is associated with functional coordination and activity in movement-related brain areas. Given the heterogeneity, small sample sizes, and predominantly indirect nature of the molecular and metabolic evidence, these mechanistic conclusions should be regarded as preliminary and hypothesis-generating pending replication in larger, comparator-controlled, and centrally-measured studies.

This review has several limitations. First, only 10 eligible studies were identified, six randomized and four non-randomized. Their Tai Chi protocols varied substantially in style, session length, and training duration (12–24 weeks). This heterogeneity prevented a meta-analysis and limits the generalisability of our mechanistic inferences. Second, most molecular findings – such as changes in HIP2 mRNA expression, tyrosine hydroxylase levels, and inflammatory cytokine profiles – come from single studies without replication and are largely peripheral/indirect rather than direct measures of central nervous system processes. These studies also used diverse biomarkers and neuroimaging methods. Consequently, the direction and magnitude of the reported associations remain uncertain. Third, no post-intervention follow-up was available for nine of the ten included studies; the single study with a 3-month follow-up had limited generalisability. This prevents assessment of long-term sustainability and limits causal inference regarding enduring neuroplastic and biomechanica changes. Meanwhile, most of the study design information was unclear (e.g., randomization and blinding procedures were not explicitly reported), introducing risks of selection bias and potential confounding factors.

Despite these limitations, our review has meaningful clinical implications for the integrated management of Parkinson’s disease. The converging, though largely indirect and preliminary, evidence from biomechanical, molecular, and neural network studies suggests that Tai Chi may be more than a general wellness activity: it appears to be a candidate multi-pathway adjunctive intervention in PD, though this interpretation requires confirmation in larger, comparator-controlled trials incorporating central biomarkers. Tai Chi has been associated with increases in tyrosine hydroxylase (a peripheral marker), reductions in pro-inflammatory cytokines (IL-1β, IL-6, TNF), and improvements in connectivity in the default mode and visual networks. These effects span both peripheral and central measures, and may plausibly complement, rather than duplicate, the action of levodopa, although this has not been directly tested. Based on this preliminary mechanistic evidence, clinicians might consider structured Tai Chi programmes – with durations and formats similar to those used in the reviewed studies (e.g., 12 to 24 weeks) – as an adjunct to standard pharmacotherapy. This may be particularly relevant for patients with mild-to-moderate PD (Hoehn and Yahr stages I–III), though the evidence base is still limited. Furthermore, the benefits of Tai Chi seem to wane after training cessation. This finding highlights the need for long-term adherence strategies and community-based maintenance programmes – a priority for future health service research.

Future research should focus on exploring the specific molecular mechanisms by which TC regulates neurotransmitters, particularly its effects on the dopamine system in PD patients, ideally using central rather than peripheral biomarkers. Additionally, using neuroimaging technology to reveal how TC impacts the functional connectivity of motor-related brain areas will be crucial, with adequately powered, comparator-controlled samples. Investigating the differential effects of various training durations and intensities on neural network adaptation is also important. These studies will provide a more scientific foundation for optimizing and personalizing TC interventions.

## Data Availability

The original contributions presented in the study are included in the article/Supplementary material, further inquiries can be directed to the corresponding author.
