# Supplementary material for: Tai Chi for improving motor symptoms in Parkinson’s disease patients: a systematic review of mechanisms
Source: Front Aging Neurosci. 2026 Aug 19;18:1883064. doi: 10.3389/fnagi.2026.1883064 (PMC13534119; doi:10.3389/fnagi.2026.1883064)
Supplement: Supplementary file 1 [file Data_Sheet_1.docx]

Supplemental material 1 Summary of studies involved.

| Reference | Study design (N) | Comparison groups | Time with PD and stage of Hoehn and  Yahr (mean SD) | Treatment regimen  (wk*dura-tion/d); length  of study | Intervention | Outcome measure | Results | Mechanism discussion |
| --- | --- | --- | --- | --- | --- | --- | --- | --- |
| 1 | DEGs were screened and analyzed using WGCNA  (N=21) | **Single group:** patients with PD who completed a 12-week Tai Chi training program. | Time with PD: 4.98 ± 3.16 years. | N/A; 12wk | Tai Chi (The type of Tai Chi and the inclusion of Qi Gong were not addressed.) | Outcome measures were collected before and after the TC Exercise:  Upregulated or downregulated proteins in peripheral leukocytes. | - RNASE7 in the blood leukocytes was upregulated. - TC influences PD immune response via the NOD-like receptor and IL-17 pathway. - positive changes in peripheral immunity, involving key genes: FOS, FOSB, JUNB, ZFP36, CAMP, and LCN2. | FOS, FOSB, and JUNB proteins activate the transcription factor AP-1. ZFP36 regulates inflammation, while the cAMP/PKA pathway enhances dopamine neuron function. LCN2 modulates neuroinflammation. They play a role in peripheral inflammation and the attenuation of inflammation induced by TC training. |
| 2 | Before-and-after study  (N=17) | **Single group:** participants with PD met online  for 60 min, 3 times weekly for 12-weeks. Biomechanical Tai Chi program with seven Yang-style forms created. | Time with PD: 7.1 ± 5.7; stage of Hoehn and Yahr: 1 - 2. | 12wk*60min | Seven Yang-style forms (The inclusion of Qi Gong was not addressed.) | Outcome measures were collected  before and after the 12-week TC intervention: Kinematic Data in the Obstacle Crossing Test (Gait speed, etc.); SLS and TUG tests. | - UPDRSIII, SLS with EO and EC scores showed minimal change pre- and post-intervention. And mean TUG time significantly improved by 1.6s. - Stride length and ankle angles saw significant increases, while toe and heel clearance and horizontal distance remained unchanged. - max AP COM displacement increased, and max ML COM-COP separation distance decreased significantly. | Enhanced muscle activation and strength, coupled with modified hip and ankle joint angles during obstacle crossing, significantly increased stride length, reflecting improved movement and posture control. TC training demonstrated low toe clearance, indicating heightened foot position awareness. Parkinson's participants exhibited improved dynamic postural stability, evidenced by decreased ML COM-COP separation and increased AP COM displacement. |
| ^3^ | Single-blinded  randomized  control trial  (N = 30) | **TC-AOT group (N=15):** a training trial consisted of  three phases. Tai Chi movements were selected from the most popular Yang style  **TPT group (N=15):** The TPT group took a stretching and flexibility exercise. | Stage of Hoehn and Yahr: 1 – 2.5. | 12wk*60min | Yang style Tai Chi (The inclusion of Qi Gong was not addressed.) | Outcome measures were collected before and after the intervention. The FCs were evaluated by seed-based correlation analysis based on the DMN, the default modes network with 18 priori seeds was selected (eg. anterior). Also, other functions were assessed using UPDRS-III, MoCA, BBS, MMSE, PDQ-39 and MiniBESTest | - UPDRS-III scores decreased, BBS, Mini-Bestest, and PDQ-39 scores increased. The TC-AOT group showed a higher MoCA score post-intervention, while the TPT group exhibited a decreasing trend. - the TC-AOT group exhibited significantly increased FCs between the dorsal medial prefrontal cortex and cerebellum crus I, between the posterior inferior parietal lobe and supramarginal gyrus, and between the temporal parietal junction and clusters of middle occipital gyrus and superior temporal. | The TC-AOT promotes early-stage PD rehabilitation outcomes by promoting brain neuroplasticity where the FCs involved in the integration of sensorimotor processing and motor learning were strengthened. And increased FCs between brain regions linked to executive attention and motor learning, including TPJ, SMG, IFG, and insula, correlated with UPDRS-III and BBS score improvements. |
| ^4^ | Randomized  control trial  (N = 90) | TC group (n=32), brisk walking group (n=31) and no-exercise groups(n=32) | Stage of Hoehn and Yahr: 1 – 2.5. | 12wk. | Tai Chi (The type of Tai Chi and the inclusion of Qi Gong were not addressed.) | Outcome measures were collected at baseline, 6 months and 12 months during one-year intervention. BBS,  UPDRS, TUG and spatial 3D  gait analysis were used to assess motor symptoms  of PD patients. Also, fMRI, plasma cytokines and metabolomics, and the blood HIP2 mRNA level were assessed. | - Participants in the Tai Chi group had better performance in BBS, UPDRS, TUG and step width. Besides, Tai   Chi was advantageous over brisk walking in improving BBS and step width.   - The improved BBS was correlated with enhanced visual network function and downregulation of interleukin-1β. - The improvements in UPDRS were associated with enhanced default mode network function, decreased L-malic acid and 3-phosphoglyceric acid, and increased adenosine and HIP2 mRNA levels. - arginine biosynthesis, urea cycle, tricarboxylic acid cycle and beta oxidation of very-long-chain fatty acids were also improved by TC training. | Enhanced brain network function, reduced infammation, improved amino acid metabolism, energy metabolism and neurotransmitter metabolism, as well as decreased vulnerability to dopaminergic degeneration may be mechanisms underlying the efects of Tai Chi training. |
| ^5^ | Randomized  control trial  (N = 195) | **TC group (N=65):** The protocol consisted of six tai chi movements, Participants engage in symmetrical and diagonal movements.  **Resistance training group (N=65):** The routine involved 8 to 10 exercises, including forward and side steps. **Stretching group (N=65):** The core activities encompassed a variety of seated and standing stretches involving the upper body. | Stage of Hoehn and Yahr: 1 – 4. | 24wk*60min | Six Tai Chi forms,2 integrated into an 8-form routine (The type of Tai Chi and the inclusion of Qi Gong were not addressed.) | The primary outcomes were changes from baseline in the limits-of-stability test (maximum excursion and directional control; range, 0 to 100%). Secondary outcomes included measures of gait and strength, scores on functional-reach and TUG tests, motor scores on the UPDRS, and number of falls. | - The TC group consistently outperformed resistance training and stretching groups in maximum excursion and directional control. - The TC group also performed better than the stretching group in all secondary outcomes and outperformed the resistancetraining group in stride length and functional reach. - TC lowered the incidence of falls as compared with stretching but not as compared with resistance training. | - TC training reduced dyskinesia by increasing the ability of the participants to adopt effective sway strategies (at the ankle or hip), engage in controlled movements with improved balance control near the limit of stability, or both. - The increase in walking speed and stride length improved symptoms of bradykinesia in PD patients. - TC emphasizes weight shifting, ankle sway, and stance variations to enhance postural control and walking ability by engaging rotational trunk movements and promoting dorsiflexion and plantar flexion strength. |
| ^6^ | Before-and-after study  (N=12) | **Single group:** patients with PD who completed a 12-week Tai Chi training program. | Time with PD: 56±13.3 months; Stage of Hoehn and Yahr: 2.3±0.78 | 12wk*60min | Eight movements of 24 simplified short forms of the Yang style TC (The type of Tai Chi and the inclusion of Qi Gong were not addressed.) | Outcome measures were collected  before and after the intervention.  participants were first evaluated for disease severity using  the UPDRS motor subscale and MMSE. Also, kinematic data measurements are taken while participants are completing the obstacle crossing task (anteroposterior and mediolateral displacement of the COP). | In the comparison of pre- and post-intervention results, the PD participants showed statistically significant improvements in all the measured COP displacements. Participants showed a 124% and 135% increase in AP and ML displacement of the COP, respectively, for both the swing and stance limbs. | TC exercise increased the magnitude of the COP displacement in the AP and ML directions, thereby improving the mechanism by which momentum is generated in the AP and ML directions in the initiation of gait and the maintenance of balance and lateral stability. |
| ^7^ | Two-arm, wait-list controlled randomized  control trial  (N=32) | **TC group (N=16)**: TC training in addition tousual healthcare;  **UC group (N=16):** usual healthcare alone. | Stage of Hoehn and Yahr: 1 – 2.5. | 6 mon*60min | The PD-specific TC program (Included mindfulness, focused attention, and mindful breathing etc.) | Change in DT gait STV from baseline to 6 months was defined, a priori, as the clinical outcome measure of primary interest. Other outcomes included: PD motor symptoms progression UPDRS, PDQ-39, executive function (Trail Making Test), balance confidence (ABC), and TUG. | - Statistically   nonsignificant improvements were observed in the TC group at 6 months in DT gait STV (TC [20.1%] vs UC [0.1%] group [effect size 0.49; P ¼.47]).   - ABC, TUG, and PDQ-39, UPDRS progression was modest and very similar in TC and UC groups. | STV serves as a valuable metric to assess cognitive-motor improvements, potentially unveiling mechanisms of improved postural control in PD patients after TC training. |
| ^8^ | Two single blinded randomized control trials  (N=45)  Project 1:  N=21  Project 2:  N=24 | Project 1:  TC Group (N=12): participated in 60-min TC  sessions for 16 consecutive weeks.  Qi-Gong meditation control (N=9):  practiced a  60-min  Qi-Gong meditation.  Project 2:  TC Group (N=15): participated in 60-min TC three times a week.  Control Group (N=9): None. | N/A | 16wk*60min | First eight movements of the Yang-style short forms (Qichong was not included) | All participants in both projects visited the laboratory both before and after the  assigned intervention period for the evaluations of their GI, gait performance and  parkinsonian disabilities. | No significant difference was observed between  groups. | Not applicable. |
| ^9^ | Before-and-after study  (N=10) | Single group: patients with PD who completed a 12-week 12 modified forms of Yang style TC. | Stage of Hoehn and Yahr: 2 – 4. | 12wk*60min | 12 modified forms of Yang style TC (Qichong was not included in the TC training.) | Outcome measures were collected  before and after the intervention. Each evaluation will collect the subjects' GRFs and analyze the COP data to compare the changes of COP trajectory variables before and after TC exercise. | The paired t-test revealed a significant difference  between before and after TC exercise in A-P and M-L  displacement of the COP of the participants(P<0.05).  TC participants showed a 122 % and 130 %  increase in A-P and M-L displacement of the COP,  respectively for both the swing and stance limbs. | participants  with PD who participated in a program of TC  exercise increased A-P and M-L displacement of the COP,  thereby improving their ability to generate momentum in  initiation of gait and maintenance of balance and lateral  Stability.  Increased COP  displacement in the M-L direction may be the result of improved coordinated action of the hip  abductor and adductor muscles. |
| ^10^ | Randomized controlled trial (N=61) | Aerobic exercise (N=20); Tai Chi exercise (N=21); Control (N=20) | Stage of Hoehn and Yahr: 1–2 (early-stage) | 12wk*60min | Yang-style short form Tai Chi (included body orientation, breathing techniques, and relaxation methods) | Blood antioxidant markers (8-OHdG, CAT, GSH, GSH-Px, GSSG, SOD, UA); cognitive function (MMSE); lower limb strength (Chair Stand); dynamic balance (8-Foot Up-and-Go); cardiorespiratory fitness (VO₂max) | Increased GSH (p=0.042), improved strength and balance, no change in VO₂max or other antioxidants. | Tai Chi exercise improves motor symptoms primarily by elevating GSH levels, enhancing antioxidant defense, reducing oxidative stress on dopaminergic neurons and muscle tissue. Unlike AE, TCE does not improve cardiorespiratory fitness; its benefits likely derive from GSH-mediated redox balance and motor skill learning (neuroplasticity). |

Abbreviations: PD, Parkinson’s disease; Tai Chi, TC; DEGs, differentially expressed genes; WGCNA, weighted gene co-expression network analysis; SLS, single leg standing; TUG, timed up and go; UPDRSIII, Unified Parkinson's disease Rating Scale – motor section; EO, Eyes Opened; EC, Eyes Closed; AP, anterior-posterior; ML, medial-lateral; COM, Center of Mass; COP, Center of Pressure; EMG, electromyography; Gmed, gluteus medius; HC, healthy controls; TFL, tensor fasciae latae; TC-AOT, Tai Chi-based action observation training; TPT, traditional physical therapy; FC, functional connectivity; DMN, default mode network; MoCA, Montreal Cognitive Assessment; BBS, Berg Balance Scale; MMSE, Mini-Mental Status Examination score; MiniBESTest, Mini Balance Evaluation Systems Test; PDQ-39, Parkinson’s Disease Questionnaire-39; TPJ, temporal parietal junction; SMG, supramarginal gyrus; IFG, inferior frontal gyrus; HIP2, huntingtin interaction protein 2; fMRI, functional magnetic resonance imaging; DT, dual-task; UC, usual care; STV, stride-time variability; ABC, ActivitySpecific Balance Confidence Scale; GI, gait initiation; GRF, ground reaction forces; 8-OhdG, 8-hydroxydeoxyguanosine; CAT, catalase; GSH, glutathione; GSH-Px, glutathione peroxidase; SOD, superoxide dismutase; UA, uric acid; GSSG, oxidized glutathione.

1.	Yang G, Dong Q, Yang H, Wang F, Chen L, Tang J, et al. Changes Observed in Potential Key Candidate Genes of Peripheral Immunity Induced by Tai Chi among Patients with Parkinson's Disease. Genes (Basel) 2022; 13(10).

2.	Law NY, Li JX. Effects of a 12-week online Tai Chi intervention on gait and postural stability in individuals with Parkinson's disease. Sports Med Health Sci 2023; 5(3): 239-44.

3.	Meng L, Wang DY, Shi Y, Li Z, Zhang JH, Lu HN, et al. Enhanced brain functional connectivity and activation after 12-week Tai Chi-based action observation training in patients with Parkinson's disease. Frontiers in aging neuroscience 2023; 15.

4.	Li G, Huang P, Cui SS, Tan YY, He YC, Shen X, et al. Mechanisms of motor symptom improvement by long-term Tai Chi training in Parkinson's disease patients. Transl Neurodegener 2022; 11(1): 6.

5.	Li FZ, Harmer P, Fitzgerald K, Eckstrom E, Stock R, Galver J, et al. Tai Chi and Postural Stability in Patients with Parkinson's Disease. New England Journal of Medicine 2012; 366(6): 511-9.

6.	Kim HD, Jae HD, Jeong JH. Tai Chi Exercise can Improve the Obstacle Negotiating Ability of People with Parkinson's Disease: A Preliminary Study. J Phys Ther Sci 2014; 26(7): 1025-30.

7.	Vergara-Diaz G, Osypiuk K, Hausdorff JM, Bonato P, Gow BJ, Miranda JG, et al. Tai Chi for Reducing Dual-task Gait Variability, a Potential Mediator of Fall Risk in Parkinson's Disease: A Pilot Randomized Controlled Trial. Glob Adv Health Med 2018; 7: 2164956118775385.

8.	Amano S, Nocera JR, Vallabhajosula S, Juncos JL, Gregor RJ, Waddell DE, et al. The effect of Tai Chi exercise on gait initiation and gait performance in persons with Parkinson's disease. Parkinsonism Relat Disord 2013; 19(11): 955-60.

9.	Kim HD, Kim TY, Jae HD, Son ST. The Effects of Tai Chi Based Exercise on Dynamic Postural Control of Parkinson's Disease Patients while Initiating Gait. Journal of physical therapy science 2011; 23(2): 265-9.

10.	Tsai CL, Chien CY, Pan CY, Tseng YT, Wang TC, Lin TK. Effects of long-term Tai Chi vs. aerobic exercise on antioxidant activity and cognitive function in individuals with Parkinson's disease. Behav Brain Res 2025; 476: 115274.
