# Supplementary material for: Tai Chi for improving motor symptoms in Parkinson’s disease patients: a systematic review of mechanisms
Source: Front Aging Neurosci. 2026 Aug 19;18:1883064. doi: 10.3389/fnagi.2026.1883064 (PMC13534119; doi:10.3389/fnagi.2026.1883064)
Supplement: Supplementary file 2 [file Data_Sheet_2.docx]

**Supplementary material 2. Central evidence table summarising all 11 included studies (new table, added in response to review).**

| **Study** | **Design (N)** | **PD stage (H&Y)** | **TC protocol** | **Comparator** | **Mechanistic outcome(s)** | **Main finding(s)** | **rating** |
| --- | --- | --- | --- | --- | --- | --- | --- |
| Yang et al., 2022 | Single-arm, before-after (N=21) | Not reported (time with PD 4.98±3.16 y) | 12 wk; style/Qigong not stated | None (single group) | Peripheral leukocyte gene expression (WGCNA/DEGs) | ↑RNASE7; altered FOS, FOSB, JUNB, ZFP36, CAMP, LCN2 — indirect immune/inflammatory signal | NOS 6/9  ROBINS-I: Serious |
| Law & Li, 2023 | Single-arm, before-after (N=17) | H&Y 1–2 | 12 wk × 60 min; Yang-style, 7 forms | None (single group) | Kinematic COP/COM, SLS, TUG | TUG improved 1.6 s; ↑AP COM displacement; ↓ML COM-COP separation | NOS 7/9  ROBINS-I: Serious |
| Meng et al., 2023 | Single-blinded RCT (N=30: TC-AOT 15 vs TPT 15) | H&Y 1–2.5 | 12 wk × 60 min; Yang-style + action-observation training | Traditional physical therapy (stretching/flexibility) | Resting-state fMRI DMN/task-related FC; UPDRS-III, MoCA, BBS, PDQ-39, MiniBESTest | ↓UPDRS-III; ↑BBS/MiniBESTest/PDQ-39; ↑FC dmPFC–cerebellum crus I, pIPL–SMG, TPJ–occipito-temporal cortex | RoB 2.0: some concerns |
| Li G et al., 2022 | RCT (N=90: TC 32 vs brisk walking 31 vs no-exercise 32) | H&Y 1–2.5 | 12 wk; style not stated | Brisk walking; no-exercise control | fMRI DMN/VN; plasma cytokines & metabolomics; HIP2 mRNA; BBS/UPDRS/TUG/3-D gait | TC > brisk walking on BBS/step width; BBS linked to ↑VN FC & ↓IL-1β; UPDRS improvement linked to ↑DMN FC, altered amino-acid/TCA metabolites, ↑HIP2 mRNA (single-study, largely peripheral/indirect evidence) | RoB 2.0: some concerns |
| Li FZ et al., 2012 (NEJM) | RCT (N=195: TC 65 vs resistance training 65 vs stretching 65) | Mild-to-moderate PD; exact H&Y distribution not reported | 24 wk × 60 min; 6/8-form Tai Chi | Resistance training; stretching | Limits-of-stability (max excursion, directional control), gait/strength, functional reach, TUG, UPDRS-motor, falls | TC > resistance training & stretching on excursion/directional control; TC > stretching on all secondary outcomes; fewer falls vs. stretching (not vs. resistance training) | RoB 2.0: Low risk |
| Kim et al., 2014 | Single-arm, before-after (N=12) | Mild-to-moderate PD (H&Y not fully specified) | 12 wk; style not stated | None (single group) | UPDRS-motor, MMSE; COP (AP/ML) during obstacle crossing | Significant ↑COP displacement (AP 124%, ML 135%, swing/stance limbs) | NOS 6/9  ROBINS-I: Serious |
| Vergara-Diaz et al., 2018 | Pilot RCT, dual-task design (N≈24; exact per-arm N incompletely reported) | H&Y not fully reported | 12 wk × 60 min, 3×/week; Tai Chi vs Qigong-meditation active control | Qigong meditation (active control) | Dual-task gait — stride-time variability (STV) | TC reduced dual-task stride-time variability, a proposed mediator of fall risk | RoB 2.0: some concerns |
| Amano et al., 2013 | RCT (control arm N=9; TC-arm N and full allocation incompletely reported) | Not fully reported | Duration not fully reported | Control (no intervention); allocation concealment unclear | Gait initiation kinematics, GRFs, COP trajectory | Inconsistent effects — TC not shown to improve gait initiation/performance in this trial | RoB 2.0: high |
| Kim et al., 2011 | Single-arm, before-after (N=10) | Not fully reported | 12 wk × 60 min; 12 modified Yang-style forms (no Qigong) | None (single group) | GRFs; COP trajectory (AP/ML) during gait initiation | Significant ↑AP and ↑ML COP displacement pre- vs. post-TC | NOS 7/9  ROBINS-I: Serious |
| Tsai et al., 2025 | RCT (N=61: TC 21 vs aerobic exercise 20 vs control 20) | H&Y 1–2 (early-stage) | 12 wk × 60 min; Yang-style short form (orientation, breathing, relaxation) | Aerobic exercise; no-exercise control | Blood antioxidant markers (8-OHdG, CAT, GSH, GSH-Px, GSSG, SOD, UA); MMSE; chair-stand; 8-ft up-and-go; VO^2^max | ↑GSH (p=0.042); improved strength & balance; no change in VO2max or other antioxidant markers | RoB 2.0: some concerns |

Abbreviations: AOT, action observation training; AP, anterior-posterior; COM, centre of mass; COP, centre of pressure; DMN, default mode network; FC, functional connectivity; GMed, gluteus medius; GRF, ground reaction force; H&Y, Hoehn and Yahr; HC, healthy controls; ML, medial-lateral; NOS, Newcastle-Ottawa Scale; RCT, randomised controlled trial; RoB 2.0, Cochrane Risk of Bias 2.0; SLS, single-leg standing; STV, stride-time variability; TA, tibialis anterior; TFL, tensor fasciae latae; TPT, traditional physical therapy; TUG, Timed Up and Go; UPDRS, Unified Parkinson's Disease Rating Scale; VN, visual network.
